# The Radiograph in Relation to the Diagnosis and Treatment of Gastric and Intestinal Lesions

**Published:** 1913-08

**Authors:** H. P. Cole

**Affiliations:** Mobile, Ala.


### THE RADIOGRAPH IN RELATION TO THE DIAGNOSIS AND TREATMENT OF GASTRIC AND INTESTINAL LESIONS.

By H. P. Cole, M. D., Mobile, Ala.

I want to open this paper by emphasizing the absolute necessity of co-operation between the Clinitician and the Roentgenologist; and also to insist that the Roentgenologist familiarize himself with the history of each case before rendering a diagnosis.

Another essential measure to follow is to take the entire field for observation, as oftentimes symptoms revealed in one locality are shown to be manifestly reflexes from some remote pathological condition in an entirely different part of the alimentary tract.

Two methods in this field of diagnosis are being used—the Flouroscopic screen and Skiagraph. At the present time we have an apparent division among our most capable men as to these two means to an end. To my mind it is positively absurd for any one to be so extreme in accepting one method without the other; for in Flouroscopic work can be found essentials not shown on the plate, and plates certainly reveal lesions that to the Flouroscope are an impossibility.

The perfection of intensifying screens has added an inestimable asset to plate work, for by this assistance instantaneous exposures are made possible, thereby obliterating defects derived from peristaltic movements.

In studying the alimentary tract the stomach naturally falls in line for first consideration.

Haudak of the Vienna school has produced a technique for this work which I consider excellent. He gives a bismuth meal and waits six hours before examination to determine the motility of the organ by position and amount of retained meal. Then after ascertaining this by screen or plate he gives a second meal for the purpose of studying the organ further.

Schlesinger describes four types of stomachs of normal function. (Picture) Hypertonic two to three hours, Orthonic three to five hours, Hypotonic four to six hours, Atonisch six to eight hours. With Grodel he shows that the differences in form are produced by the differences in tone of the musculature.

To quote Holzknecht: "For types three and four a delay of six hours will be normal, whereas for type one it would indicate some obstruction of the pylorus.

Taking into consideration the foregoing, I beg leave to show a picture made by Haudak as to the position of the residue in relation to the umbilicus. (Picture) (a) Atony, spasm of pylorus or slight stenosis. (b) Broad extensive residue due to incompensative stenosis. (c) "Snail Form"—Residue far to the left by shrinkage of lesser curvature, due to callous ulcer. (d)

Residue displaced to left, margin well defined and jagged; no displacement of pylorus; tumor of pylorus.

In all cases where any pathological lesion is suspected a lateral view should be taken to show the wall involved.

Hour glass contraction of the stomach is often met with and the first thing connected with this condition by the average man will be a malignancy. Of course malignant tumors cause this manifestation, but Martin, Meckel, and Saake describe cases of this nature found from congenital formation.

Cicitricial contraction of a healing gastric ulcer in numerous cases produces well developed hour glass contraction.

I have seen the conditon exist under Flouroscopic examination revealing a typical contraction of this type, and while yet on the screen the spastic musculature suddenly relaxing gave the natural formed organ. Had a plate only been made you can readily see the source of error, hence the necessity of a combined technique.

Perforating gastric ulcers usually occurring on the lesser curvature is a condition often found by the Roentgenologist. The liver forms the outer wall and a pouch resembling a diditiculum is revealed.

The pyloric end of the stomach is the part of greatest importance in its study. Dr. Cannon describes a so-called Myenteric reflex as follows: "Stimulation of the mucous membrane of the alimentary tract at any point causes a contraction of the musculature above, and relaxation below the point of stimulation. In the pylorical region the stimulation is hydrochloric acid. When during the process of digestion hydrochloric acid is liberated, and remains uncombined with albumen, then the Myenteric reflexes act so as to cause a muscular contraction above the stimulating antral mucous membrane and relaxation below (that is, the pyloric sphincter will relax and cause a jet of chyme to enter the duodenum). As soon as the free acid strikes the mucous membrane of the duodenum another similar reflex is set in motion which causes similarly a contraction of the pyloric sphincter above and a dilatation of the rest of the duodenum below. This contraction of the pylorus will persist so long as there is free hydrochloric acid in the duodenum or until the secretion of alkaline intestinal juices are enough to neutralize it. Then the presence of hydrochloric acid on the stomach side of the pylorus will cause it to apen and a similar series of events follow."

Drs. George and Gerber go further and say that any abnormality in the wall to impair contact with the agent of stimulation creates a relaxation and causes very rapid evacuation. The lesion being in the duodenum, or where there is lack of acid due to carcinoma of the stomach, the relaxation of the pylorus will also create rapid emptying.

I have seen many stomachs of apparent normal position and without lesion of any type perceptible give the patient great pain, the cause to my mind being rapid evacuation of acid contents (due to chronic congestion of the pyloric mucosa) into the alkali duodenum and to such an extent as to prevent neutralization. This I believe creates an irritation in the duodenum that unless stopped will result in ulceration or adhesion of a periodical type. I have seen cases recently where X-ray findings gave very rapid evacuations and one was clinically by some pronounced carcinoma. There was no indication from the skiagraph of this condition and I attribute the trouble to a persistent acidified duodenum. The patient was treated accordingly and relieved.

Dr. Cole, the originator of "Serial Radiography," has given us a technique by which duodenal ulcer can in a great number of cases be revealed. He takes a series of 12 plates in the limited time of 20 seconds. He lays great stress on the shape of the first part of the duodenum commonly called the "Cap" and a persistent irregularity in all the plates will if not revealing a lesion plainly indicate an adhesion.

Should the cap not be revealed by an antero-posterior exposure a lateral view should be made as the pyloric end of the stomach might have obstructed the view of the duodenum.

Dr. George has demonstrated the necessity of this procedure (lateral exposure) by showing the cause of some obstruction in the lumen to be due to ulcer or new growth not shown on antero-posterior view.

The gall bladder region presents a great field for study by the Roentgenologist, and numerous cases of the so-called "gall stone" are shown to be adhesions in this locality.

The duodeno-jejunal juncture often presents a pathological condition from acute angulation.

Dilated duodena can easily be detected if there be constriction in the distal portion of the duodenum, but if this be absent the Bismuth meal will hardly bring out the true extent of the dilation. Dr. Cole has invented quite a unique plug for the duodenum. It is an inflatable rubber bag with a shot attached, and connected by a tube long enough to reach through the mouth. The air is removed from the bag which is then placed in the stomach through a larger tube and when the weight of the shot carries the bag into the duodenum it is inflated and the desired occlusion of lumen is attained, then a Bismuth meal of liquid type is given and a true outline of the duodenum can be screened or skiagraphed.

Sometimes adhesions in the gall bladder region will cause the shadow produced by the pylorus to be pulled high up and to the right of the normal position. This point must be noted closely, as the condition will decrease the emptying power of the stomach and oftentimes manifest clinical symptoms of a more serious nature.

Acute angulations are seldom found in the small intestines below the duodeno-jejunal juncture except in the ileo-caecal region where the Lane Kink is not an uncommon manifestation. All cases manifesting clinical symptoms of appendicitis should be skiagraphed to eliminate this abnormality so well described by Mr. Lane.

The points of election of the large intestines for study by the X-ray are the Caecum, the Hepatic and Spleenic flexures and the Sigmoid regions.

Of course malignancies and diverticuli might exist along any part of the tract and are readily shown by this means of examination. Where any apparent occlusion or retardation of the Bismuth meal is found by a skiagraph, a fluoroscopy should be made to ascertain the nature of peristaltic movement and also a sterioscopic skiagraph made to study the part in perspective in order that the relative position of the bowels might be shown.

Ptosis of the colon might and does exist without manifesting the slightest abnormality clinically, and the so-called double barreled colon has come under my observation in numerous cases in perfectly healthy individuals without even constipation.

However, where this condition produces angulation of a nature so acute as to prevent function and lay the foundation for pari-colitis resulting in adhesion between the two barrels, surgical intervention is the only means of relief.

Pfahler has recently described gaseous pockets caused from acute angulations of the flexures and in the elongated sigmoid—where the distended bowel pressing on some other organ manifested symptoms as if though produced directly by the organ itself. The usual locality for this trouble is found in the spleenic flexure which presses on the stomach and produces typical gastric symptoms. He reports cases of angulated elongated sigmoid creating similar symptoms.

The best way to study the colon is by means of Bismuth enemas and flouroscopic examination during the retrograde peristalsis from the rectum to the caecum. This requires from 10 to 20 minutes and if the patient is protected by a 1 m. m. thickness or a layer of chamois skin, as a filter to the rays, very little danger of a burn is chanced. Where any indication of obstruction to the progress of the enema is shown, a skiagraph should be made to study for manifestation of a lesion, and should it be in the region of the flexures a stereoscopic view for perspective examination is quite an advantage.

I want to close by saying that I consider negative findings in the viscera of paramount importance, as elimination of conditions perhaps clinically revealed might avoid many unnecessary operations and suggest other fields for cause.

